# Synergy Effect of Combining Fluorescence and Mid Infrared Fiber Spectroscopy for Kidney Tumor Diagnostics

**DOI:** 10.3390/s17112548

**Published:** 2017-11-05

**Authors:** Andrey Bogomolov, Valeria Belikova, Urszula J. Zabarylo, Olga Bibikova, Iskander Usenov, Tatiana Sakharova, Hans Krause, Olaf Minet, Elena Feliksberger, Viacheslav Artyushenko

**Affiliations:** 1 Art Photonics GmbH, Rudower Chaussee 46, 12489 Berlin, Germany; ab@globalmodelling.com (A.B.); usenov@tu-berlin.de (I.U.); ts@artphotonics.de (T.S.); ef@artphotonics.de (E.F.); sa@artphotonics.com (V.A.); 2 Laboratory of Multivariate Analysis and Global Modeling, Samara State Technical University, Molodogvardeyskaya 244, 443100 Samara, Russia; valerya.belickova@yandex.ru; 3 Medical Physics & Optical Diagnostics, CC6 Campus Benjamin Franklin, Charité Universitätsmedizin Berlin, Hindenburgdamm 30, 12203 Berlin, Germany; urszula.zabarylo@charite.de (U.J.Z.); olaf.minet@charite.de (O.M.); 4 Research-Educational Institute of Optics and Biophotonics, Saratov National Research State University, Saratov 410012, Russia; 5 Technical University of Berlin, Institute of Optics and Atomic Physics, 10623 Berlin, Germany; 6 Deptartment of Urology, Charité Universitätsmedizin Berlin, Charitéplatz 1, 10117 Berlin, Germany; hans.krause@charite.de

**Keywords:** mid infrared spectroscopy, fluorescence spectroscopy, synergy effect, fiber probe, joint data analysis, cancer diagnostics

## Abstract

Matching pairs of tumor and non-tumor kidney tissue samples of four patients were investigated ex vivo using a combination of two methods, attenuated total reflection mid infrared spectroscopy and fluorescence spectroscopy, through respectively prepared and adjusted fiber probes. In order to increase the data information content, the measurements on tissue samples in both methods were performed in the same 31 preselected positions. Multivariate data analysis revealed a synergic effect of combining the two methods for the diagnostics of kidney tumor compared to individual techniques.

## 1. Introduction

Optical spectroscopic analysis is rapidly disseminated in the clinical domain. As a new diagnostic modality, it offers unique opportunities for a label-free investigation of tissue samples at the molecular level that helps to identify various diseases. The possibility of non-invasive analysis of the tissue cellular structure capable of detecting diagnostically relevant abnormalities turns spectroscopy-based methods into a novel approach to clinical diagnostics. The so-called “spectral histopathology” has developed over the last few years as an ancillary tool for classical histopathology using surgical biopsy samples for the tissue examination [1,2]. The routine procedures of clinical histopathology are complicated and time-consuming. Besides, this diagnostic method strongly relies on the analyst’s professional qualification, and is hence prone to a human error. These facts demonstrate the need for a rapid and more objective alternative to classical histopathology.

According to the mortality statistics reported by the American Cancer Society in 2016, kidney cancer is one of the 10 most common cancer types [3] and clear cell renal cell carcinoma (cСRCC) is the main modification of kidney cancer. Renal cell carcinomas comprise approximately 3% of adult malignancies worldwide and 90%–95% of neoplasms arising from the kidney. The only known curative treatment of this cancer type is a surgical resection [4,5]. Spectral histopathology is often considered as a potential approach to revealing tumor cells in the tissue during a surgical operation.

Fluorescence spectroscopy is one of the most established optical diagnostic methods that is successfully used for cancer imaging, e.g., for the delineation of a margin between normal and cancerous tissue [6,7]. Extremely high sensitivity to specific molecules including some known cancer biomarkers [8] called fluorophores is the main analytical advantage of the fluorimetric analysis. Less sensitive, but highly selective mid infrared (MIR) spectroscopy is capable of recognizing various organic molecules based on the fundamental vibration frequencies of their functional groups and the quantification of the respective mixture constituents [9]. This chemically informative spectroscopic technique is increasingly used for the investigation of various biological materials [10]. Recent studies demonstrated the significant diagnostic potential of MIR spectroscopy for various types of human cancer [11,12,13,14].

Both fluorescence and MIR spectroscopic measurements of tissue can be performed through fiber-based probes. The main advantages of using fiber spectroscopic methods are their being non-destructive and non-invasive, as well as avoiding the use of extrinsic contrast-enhancing agents. Application of the optical fiber approaches for the determination of the tissues affected by a tumor has been demonstrated ex vivo for the brain [15], colon [16,17], and skin [18]. Recently, it has been shown that a combination of spectroscopic methods can increase the effectiveness of cancer detection [15,19]. The majority of reported multi-modal spectroscopic systems are presented by a combination of fluorescence and diffuse reflectance spectroscopy methods [20,21,22].

The present study is a part of a larger research project, the main purpose of which is the development of new optical techniques for tumor margin identification that could be subsequently transformed into a clinical method and tool. The authors’ recent work from this series [23,24] was aimed at the development and testing of a near infrared (NIR) sensor for the diagnostics of kidney tumors based on four light-emitting diodes (LEDs). In the present study, we report a synergic effect of using MIR and fluorescence spectroscopy simultaneously, in distinguishing healthy and malignant tissue samples of the human kidney obtained from an operative cancer treatment. Diagnostic capabilities of fluorescence and MIR spectroscopy have been investigated using matching pairs of normal and malignant biopsy samples of a few patients with kidney cancer. Special attention was paid to a joint analysis of spectra taken at the same sample positions in order to prove a hypothetical advantage of their possible integration within the same analytical instrument.

## 2. Materials and Methods

### 2.1. Sample Preparation

**Figure 1:**
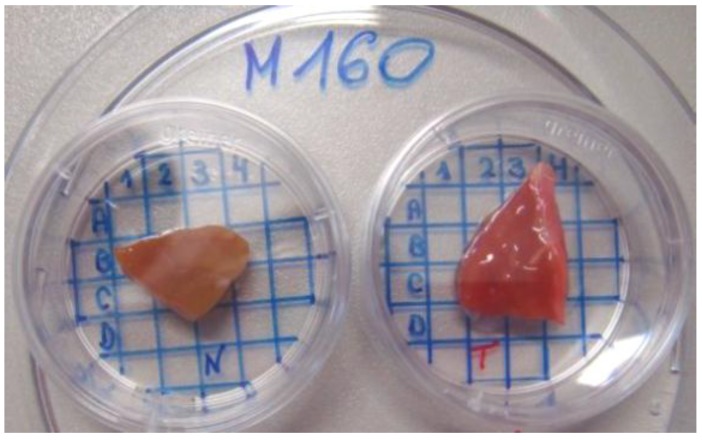
Renal biopsy of patient 160: healthy (**left**) and tumor (**right**) tissue.

Eight unstained cryo biopsies after nephrectomy (matched pairs of tumor and non-tumor tissue of the same kidney) were obtained from Department of Urolgy at Charité Universitätsmedizin Berlin (Germany). The institutional ethics committee approved the sampling and further investigation of renal tissues (ethical approval number EA1/134/12). The samples typical thicknesses were from 5 to 10 mm, in accordance with the common histopathological practice, since larger specimens could not be shock-frozen as required for the clinical investigation. All tumor samples were of the predominant cCRCC subtype. According to the Fuhrman nuclear grading system (FNG) [25], all tumor samples were classified as low-grade G1 (round well-differentiated nuclei) or intermediate grade G2 (larger nuclei with slightly irregular contours and nucleoli). Tumors were staged according to the Union for International Cancer Control Tumor-Node-Metastasis (UICC-TNM) criteria [26]. The biopsies represented four male patients: M149 (56 years old/tumor grade G1/staging pT1b), M151 (69/G2/pT3a), M160 (47/G2/pT2b), and M144 (62/G2/pT3a). The staging describes sizes of the tumor and its infiltration into the surrounding organs. Within staging classes, tumor extension increases from pT1 to pT3. Prior to measurements, tissue samples were thawed for 5 min at room temperature. A typical view of the patient biopsies is presented in Figure 1. Some further details on sample preparation can be found in previous publications by the authors [23,24,27].

### 2.2. Spectroscopic Measurements

**Figure 2:**
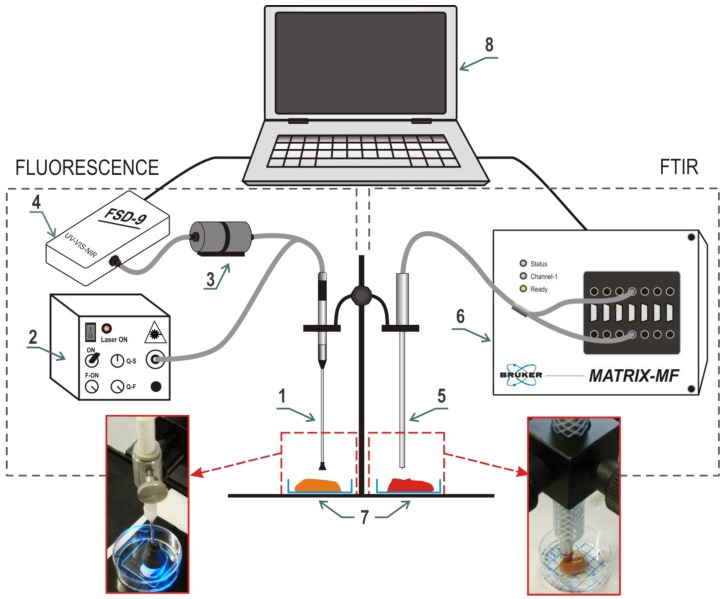
Experimental setups for fluorescence (left) and mid infrared (MIR) (right) spectral measurements: 1—fluorescence probe; 2—laser light source; 3—cut-off fluorescence filter; 4—fluorescence spectrometer; 5—attenuated total reflection MIR probe; 6—MIR spectrometer; 7—samples; 8—computer.

MIR measurements were performed using a Matrix MF (Bruker Optik GmbH, Ettlingen, Germany) spectrometer equipped with a mercury-cadmium-telluride (MCT) detector cooled by liquid nitrogen. Spectra were acquired in contact with the tissue using a polycrystalline infrared (PIR) fiber-based attenuated total reflection (ATR) probe with a silica crystal on top (art photonics GmbH, Berlin, Germany) optimized for the fingerprint region. Sterile 0.9% sodium chloride aqueous solution was used as a background (to obtain the reference spectrum). MIR spectra were obtained at the resolution of 8 cm^−1^ at 64 scans. Typical spectrum acquisition time at these settings was about 46 s. Some further details on MIR spectroscopic investigation of kidney samples have been published before [27]. Experimental setups are presented in Figure 2.

Fluorescence by cancer and normal tissues was excited at 473 nm using a 25-mW laser (art photonics GmbH, Berlin, Germany) through a needle-shaped probe by art photonics containing an aluminum-coated 400 μm core detection fiber surrounded by 13 silica illumination fibers having a diameter of 100 μm. To protect the measurement against ambient light, the probe tip was surrounded by a black plastic shield, which also provided a fixed distance to the sample of about 2 mm. The measurement was performed through a thin (0.5 mm) quartz glass covering the sample to avoid its direct contact with the probe (Figure 2). An FSD-9 mini-spectrometer (art photonics GmbH, Berlin, Germany) was used to collect fluorescence spectra in the 200–1080 nm range with optical resolution of a few nm (spectral points were sampled at 0.26 nm). A refocusator by art photonics with a FEL0500 long pass optical glass filter (Thorlabs Inc., Newton, NJ, USA) with a cut-off wavelength of 500 nm was used to suppress the signal of back-scattered illumination light in the fluorescence spectrum (element 3 in Figure 2). Spectrum acquisition times were adjusted for each new measurement position in order to keep the maximum spectrum intensity within the optimality region of the spectrometer above 30000 counts (more than 50% of the upper intensity limit). The interval of acquisition times was 125–2000 ms and the measurement was predominantly longer for tumor biopsies in comparison to the normal tissue. Higher integration times were avoided, even if the maximum spectrum intensity was lower than optimal. All repeated measurements at the same position were performed with the same acquisition time.

Three replicated measurements in each pre-selected position were made by either spectroscopic method. The repeated measurements were performed after the re-focusing of the probe on a sample using a moving probe grip.

### 2.3. Data Analysis

Multivariate data analysis including principle component analysis (PCA) [28] and partial least-squares discriminant analysis (PLS-DA) [29] was performed using TPT-cloud (www.tptcloud.com), a web-based chemometrics software by Global Modelling (Aalen, Germany) and Samara State Technical University (SSTU, Samara, Russia), and Interval Selection Toolbox (SSTU) for Matlab^TM^ (MathWorks, Natick, MA, USA). Model validation was performed by means of segmented cross-validation using the data in unique measurement positions as segments.

Second derivative algorithm by Savitzky-Golay [30] was used with the following options adjusted in preliminary data analysis: second-order of polynomial and smoothing window width of 25 points. Prior to the concatenation, appropriately reduced and preprocessed MIR and fluorescence spectra were normalized. Two normalization methods were alternatively used: standard normal variate (SNV) algorithm transforming individual spectra to the unit vector length or weighting of the variable vectors by their inverse standard deviation. The latter method in combination with subsequent mean centering is usually referred to as autoscaling (AS).

In PLS-DA modeling, cancer was conventionally considered as “positive” and health as “negative” test results, numerically coded as 1 and 0, respectively. The numbers of true positives (*TP*), false positives (*FP*), true negatives (*TN*), and false negatives (*FN*) in the prediction as well as percent accuracy *%Ac* = (*TP* + *TN*)/(*TP* + *FP* + *TN* + *FN*), sensitivity *%Sn* = *TP*/(*TP* + *FN*), and specificity *%Sp* = *TN*/(*FP* + *TN*) values were used to measure discrimination quality [31].

Segmented cross-validation (CV) and random-subset validation (RSV) were used to estimate the number of latent variables (LVs) in the PCA and PLS-DA models and to characterize the model performances in prediction. The CV segments were formed by repeated measurements in different sample positions. In the RSV method, a random subset of 14 spectra was excluded at the modeling stage to be used for an independent prediction. The subset size of about 15% of the whole data was chosen as a compromise between representativeness of the residual training data and soundness of the test-set prediction statistics. To compensate for the random factor in the modeling statistics, the procedure was repeated 1000 times and cumulative numbers of *TP, FP, TN*, and *FN* were used to calculate the *%Sn, %Sp*, and *%Ac* values. A large number of iterations of the subset selection-modeling-validation cycle was necessary to assure the statistics convergence to constant values independent of a particular subset. The optimal number of LVs in the PLS-DA models was determined from the CV data.

## 3. Results and Discussion

Selection of appropriate spectroscopic techniques and their combinations followed by the development of analytical methods and instruments consists of multiple measurement steps involving clinical samples. Considering the specific nature of the samples, their limited availability, and the ethic aspects of their use, intermediate studies (e.g., method comparison) necessary to make informed development decisions should be based on a possibly small set of patients. Obtaining necessary samples is additionally complicated by the requirement that pairs of cancer and healthy tissue biopsies should belong to the same kidney after nephrectomy, i.e., full excision of the organ. This operation takes only about 30% of cancer surgical treatment cases at Charité [32]. At partial excisions, the healthy tissue should be possibly preserved, and hence, noncancer samples are typically absent. In order to enhance the data information content, spectral measurements of eight available biopsy samples were performed in 31 preselected sample positions (from three to five positions on each individual biopsy) coded using a coordinate grid, as shown in Figure 1. The experiment was designed to include the main practically relevant variabilities, as required for the data realism and consistency. Therefore, the resulting spectra comprised both intra- and inter-sample variability and was well-suited for a joint analysis of MIR and fluorescence data.

**Figure 3:**
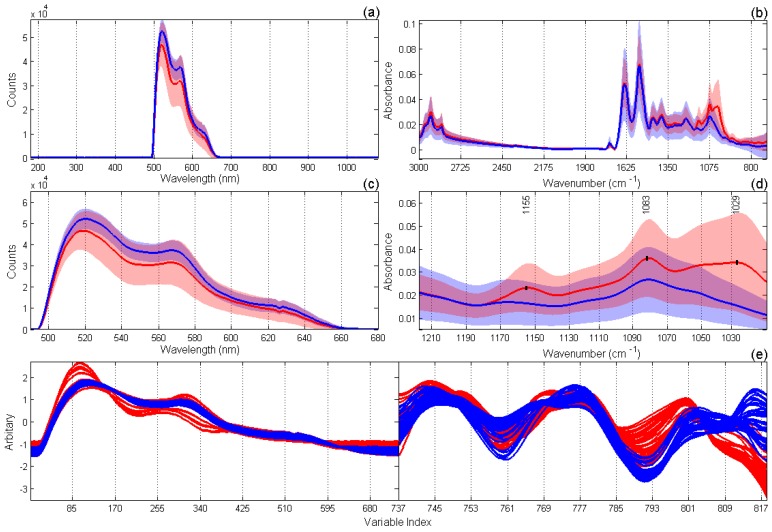
Spectral data: (**a**) raw fluorescence spectra; (**b**) raw mid infrared (MIR) spectra; (**c**) fluorescence spectra in the region of 490–680 nm; (**d**) MIR spectra in the region of 1220–1010 cm^−1^; and (**e**) concatenated dataset of preprocessed fluorescence (left side) and MIR (right side) spectra. The following preprocessing was applied before data concatenation: standard normal variate (SNV) for fluorescence data and Savitzky-Golay second derivative followed by SNV for MIR spectra. Red and blue colors correspond to tumor and normal tissue samples, respectively. The curves and the surrounding colored regions in (**a**–**d**) represent the mean spectra and the standard deviation intervals of the respective data.

The resulting individual datasets included 92 spectra and 82 (MIR) or 736 (fluorescence) variables. The raw spectral data are presented in Figure 3a,b.

MIR spectra were obtained in the full range of 3000–700 cm^−1^ (Figure 3b). It can be noticed that probably the most significant spectral difference of the cancer tissue is related to the presence of a larger peak with an average intensity maximum at 1083 cm^−1^ (Figure 3b,d) caused by symmetric stretching vibrations of the ionized ${\mathrm{PO}}_{2}^{-}$ group [33]. Phosphate ${\mathrm{PO}}_{2}^{-}$ groups with stretching vibration in these regions mainly originate from the phosphodiester groups of cellular nucleic acids, membrane phospholipids, and partially from protein (amide III). Higher intensity of this band assumes an increased concentration of the nucleic acids in the tumor tissue due to a higher proliferation rate of the tumor cells [34]. A weak spectral difference associated with the phosphate was found at around 1240 cm^−1^ (Figure 3b) and assigned to the asymmetric stretching vibrations of ${\mathrm{PO}}_{2}^{-}$ [34,35]. Also, an increased level of glycogen manifested in a stronger absorption at 1026–1030 cm^−1^ (the average maximum at 1029 cm^−1^) was observed for the malignant tissue due to an activation of the glycolysis. These significant changes in the carbohydrate metabolism are characteristic of the renal cell carcinoma tissues. Another carbohydrate-related absorption band at 1155 cm^−1^, which is stronger in malignant kidney, was assigned to the C–O and C–OH stretching vibrations [34]. An increase of absorption around 1155 cm^−1^ observed for the malignant tissue originated from the C–OH stretching mode by amino acid (threonine, tyrosine, and serine) residues in the cell proteins. In general, the metabolism alteration of carbohydrates and lipids definitely belongs to the malignant modification process of cCRCC. The group of peaks between 3000 and 2800 cm^−1^ associated with the vibrations of aliphatic residuals did not reveal any noticeable correlation with the diagnosis. Therefore, only the region of 1220–1010 cm^−1^ containing the most relevant signals (Figure 3d) was taken for the PLS-DA modeling. Similarly, fluorescence spectra were reduced to the signal region of 490–680 nm. 

Regarding the fluorescence signals of renal biopsies, the most important fluorophores contributing to the peaks at 520 nm, 560 nm, and 630 nm are supposed to be flavin adenine dinucleotide (FAD), collagen, and porphyrins, respectively. Several studies have reported the interstitial expression of collagen types I and III in renal cell carcinoma [36]. Differences in fluorescence signals between tumor and normal samples may come from the changing ratio of their composition, but can also be attributed to the appearance of at least one additional fluorophore [37].

As it follows from the assignment of spectral features, the methods of fluorescence and MIR spectroscopy are capable of delivering complementary chemical information, and are therefore suitable for a joint analysis.

**Figure 4:**
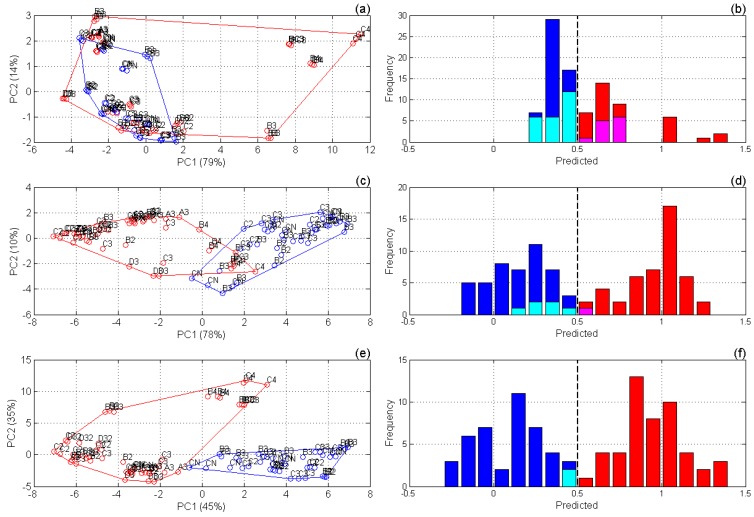
Principal component analysis (PCA) and partial least-squares discriminant analysis (PLS-DA) models (segmented cross-validation results): (**a**,**c**,**e**) score plots of PCA models and (**b**,**d**,**f**) frequency histograms of PLS-DA-predicted values for: (**a**,**b**) fluorescence spectra corrected by standard normal variate (SNV), (**c**,**d**) SNV-corrected second derivative MIR spectra, and (**e**,**f**) concatenated dataset. In (**a**,**c**,**e**): red and blue colors designate tumor and normal tissue samples, respectively; labels designate measurement positions on the sample; percent variances explained by the corresponding principal components are shown in brackets on the axis labels. In (**b**,**d**,**f**): blue, cyan, red, and magenta colors designate true negatives (*TN*), false negatives (*FN*), true positives (*TP*), and false positives (*FP*), respectively.

Preliminary PCA of the raw spectral data has shown a much better tumor/normal class separation in the case of combined dataset, as observed in the scores plots in Figure 4a,c,e. 

The following approach to the data preprocessing was used to rank the discrimination abilities of individual spectroscopic techniques and their combinations. Individual techniques were tested with different preprocessing methods prefacing PLS-DA modeling: no preprocessing or SNV for fluorescence data and no preprocessing, SNV, second derivative (2D), or 2D + SNV (2D followed by SNV) for MIR data. In the case of spectra concatenation, both data parts should be normalized in order to standardize their scales, and thus, to minimize the model bias. Therefore, in the case of combined data, the “no preprocessing” method was not considered at all. Both data parts in this case were SNV-corrected or autoscaled (Section 2.3). The best method was chosen based on the accuracy (*%Ac*) statistics of calibration and validation. The results presented in Table 1 enable a comparison of the diagnostic efficiency of two separate spectroscopic techniques as well as their combination after the individually adjusted data preprocessing method chosen from the list of the most efficient algorithms.

Calibration, CV, and RSV statistics show similar numbers and the method ranking stays mainly the same. This similarity is an indicator of the absence of model overfitting, at least for the present dataset. Overfitting is a common analytical risk to be considered, especially when the data volume is limited. Although the present dataset seems adequate for the method comparison, the practical diagnostic models should be built on a much larger number of patients to cover all possible variabilities. The modeling statistics in that case can be less optimistic. It is also remarkable that both individual and combined discrimination models required only two LVs. The model simplicity that stays unchanged in spite of the growing number of variables and hence data complexity is another sign of the method robustness. The statistics in Table 1 show that CV with the segments formed by measurement positions was the most conservative and hence the most straightforward validation strategy. Every iteration of the segmented CV simultaneously excludes similar spectra of the same sample positions. RSV is another good option for relatively small datasets that combines the features of both independent validation and CV. 

Fluorescence data by itself show generally lower discrimination capabilities than MIR spectroscopy. The respective CV accuracies were 61% against 92% for the best preprocessing techniques (bold font in Table 1). SNV correction of the fluorescence spectra results in some improvement of the respective model performance. In contrast, raw spectra produce the worst discrimination statistics of the MIR spectroscopic model. The best preprocessing for the individual MIR method was found to be 2D + SNV, which resulted in a noticeably better prediction than SNV or second derivative methods alone (Table 1). Misclassified measurements in the MIR-based model are predominantly presented by false negatives, i.e., non-recognized tumor (Figure 4d), which is an undesired trend. 

Concatenation of the fluorescence spectra with second derivative MIR spectra followed by SNV normalization of both data parts leads to a remarkable improvement. CV of the optimal PLS-DA model built on the combined spectral data (Table 1 and Figure 4f) resulted in only two misclassifications of 92 measurements, which corresponds to a 98% accuracy. Other preprocessing techniques and their combinations were also checked, but the model performance was significantly lower (Table 1). 

The role of an appropriate preprocessing for the whole success of analysis is high. Second derivative preprocessing was necessary to compensate for unavoidable baseline variations observed in MIR spectra (Figure 3b). As the fluorescence spectra were acquired with different acquisition times, their SNV normalization was necessary to remove the absolute intensity effect and to emphasize the differences in the spectral shape. For the data weighting that accompanies their concatenation, SNV correction worked out to be a much better normalization algorithm than autoscaling. 

Increasing use of multi-spectral techniques is a distinct trend in modern qualitative and quantitative analysis [38]. However, the simple addition of any spectroscopic method does not generally result in synergy. For instance, a combination of MIR [39] or NIR [40] with Raman spectroscopy did not bring any accuracy gain of the target component determination. At the same time, the latter combination was profitable for another analyte [40]. An evident gain of merging two optical techniques observed in the present study allows for a reasonable suggestion about the difference of respective cancer biomarkers predominantly revealing themselves in MIR and fluorescence spectra, which also follows from the above given spectral interpretation. Also considering the size of effect, it can hardly be explained by simple mutual compensation of the measurement errors due to the combination of techniques.

Due to its small penetration depth (0.5–2 μm) at the MIR wavelengths, ATR spectroscopy is a superficial measurement technique that mainly works at the cellular level. In contrast, fluorescence signals can be collected from a depth of up to a few millimeters, as corresponds to the higher penetration ability of the visible laser light. The depth can also be affected by the scatter, depending, in its turn, on tissue morphology. Working depth is therefore an additional factor making the information delivered by both methods complementary.

Joint analysis of MIR and fluorescence spectra of the same objects is new; neither medical nor industrial applications of this combination are heard of. This lack of research is accounted for by experimental differences making compatible measurements of the same solid sample by both methods problematic. In the present study, the compatibility was possible due to the application of a PIR-based ATR probe, thus enabling spectroscopic MIR and fluorescence measurements at the same sample point.

The experimental data discussed in this section can be accessed using the links provided in Appendix A.

## 4. Conclusions and Outlook

The observed synergic gain of combining fiber-based ATR MIR and fluorescence spectroscopy of kidney cancer provides a motivation for the further development and improvement of this joint method for in vitro diagnostics. For clinical usage, both techniques should be seamlessly integrated within the same analytical method and probe. The main requirement and, at the same time, the main challenge of the probe unification is to provide a simultaneous measurement of exactly the same point at the sample surface.

Significant effort should be aimed at the accumulation of statistically representative data to provide the robustness of the multivariate discriminating model, i.e., its resistance to all possible variations of the sample tissue. The method extension to different organs and cancer types is another important direction of its development.

A separate challenge to be addressed in our subsequent studies is the fair method comparison. The need for a formalized approach to the comparison of spectroscopic and modeling techniques follows from an extremely wide variability of the tissue samples on the one hand, and from the diversity of statistical criteria on the other. The sum of ranking differences (SRD) method [41,42] provides a necessary platform for an unbiased method discrimination and validation [43] based on a representative clinical data including multiple datasets with numerous patients and different measurements sites. 

## Figures and Tables

**Table 1:** Comparison of spectroscopic methods for kidney cancer diagnostics; two latent variables (LVs) were used in all models.

Method	Preprocessing	*TP*	*FP*	*TN*	*FN*	*%Ac*	*%Sn*	*%Sp*
*Calibration* ^1^								
Fluorescence	none	37	20	21	14	63	73	51
	SNV ^2^	**32**	**12**	**29**	**19**	**66**	**63**	**71**
MIR	none	38	1	40	13	85	75	98
	SNV	42	2	39	9	88	82	95
	2D ^3^	45	4	37	6	89	88	90
	2D + SNV	**49**	**0**	**41**	**2**	**98**	**96**	**100**
Fluorescence | MIR	AS ^4^ | AS	39	13	28	12	73	76	68
	AS | 2D + AS	44	8	33	7	84	86	80
	SNV | SNV	48	0	41	3	97	94	100
	SNV | 2D + SNV	**51**	**0**	**41**	**0**	**100**	**100**	**100**
*Cross-validation* ^5^								
Fluorescence	none	32	22	19	19	55	63	46
	SNV	**27**	**12**	**29**	**24**	**61**	**53**	**71**
MIR	none	38	3	38	13	83	75	93
	SNV	42	4	37	9	86	82	90
	2D	45	5	36	6	88	88	88
	2D + SNV	**45**	**1**	**40**	**6**	**92**	**88**	**98**
Fluorescence | MIR	AS | AS	35	15	26	16	66	69	63
	AS | 2D + AS	37	9	32	14	75	73	78
	SNV | SNV	47	0	41	4	96	92	100
	SNV | 2D + SNV	**49**	**0**	**41**	**2**	**98**	**96**	**100**
*Random-subset validation* ^6^								
Fluorescence	none					61	70	50
	SNV					**65**	**61**	**70**
MIR	none					84	75	95
	SNV					88	83	94
	2D					89	87	91
	2D + SNV					**95**	**92**	**99**
Fluorescence | MIR	AS | AS					71	75	66
	AS | 2D + AS					81	83	80
	SNV | SNV					96	94	100
	SNV | 2D + SNV					**99**	**98**	**100**

^1^ Prediction and training on the full dataset; ^2^ Standard normal variate; ^3^ Savitzky-Golay second derivative; ^4^ Autoscaling; ^5^ Segmented CV with 31 segments formed by the measurement positions; ^6^ Subset (15%) of the full data at 1000 iterations.

## Appendix A. Source Data

Fluorescence spectra: https://tptcloud.com/data/view/4904

MIR spectra: https://tptcloud.com/data/view/4905

Concatenated spectra after optimal preprocessing (Table 1): https://tptcloud.com/data/view/4902
